# Efficacy and safety of renal denervation in addition to pulmonary vein isolation for atrial fibrillation and hypertension—Systematic review and meta‐analysis of randomized controlled trials

**DOI:** 10.1002/joa3.12353

**Published:** 2020-04-27

**Authors:** Raymond Pranata, Rachel Vania, Sunu Budhi Raharjo

**Affiliations:** ^1^ Faculty of Medicine Universitas Pelita Harapan Tangerang Indonesia; ^2^ Department of Cardiology and Vascular Medicine Faculty of Medicine Universitas Indonesia National Cardiovascular Center Harapan Kita Jakarta Indonesia

**Keywords:** atrial fibrillation, atrial fibrillation recurrence, catheter ablation, pulmonary vein isolation, renal denervation

## Abstract

**Introduction:**

This systematic review and meta‐analysis aimed to assess the latest evidence on the use of renal denervation (RDN) + pulmonary vein isolation (PVI) compared to PVI alone for treating atrial fibrillation (AF) with hypertension.

**Methods:**

A systematic literature search from several electronic databases was performed up until January 2020. The primary outcome was AF recurrence defined as AF/atrial flutter (AFL)/atrial tachycardia (AT) ≥30 seconds at 12‐month follow‐up and the secondary outcome was procedure‐related complications.

**Results:**

There were 568 subjects from five studies. AF recurrence was 90/280 (32.1%) in the RDN + PVI group and 142/274 (51.8%) in the PVI group. RDN + PVI was associated with a lower incidence of AF recurrence (RR 0.62 [0.51, 076], *P* < .001; *I* ^2^: 0%). Pooled analysis of HR showed that RDN + PVI was associated with reduced AF recurrence (HR 0.51 [0.38, 0.70], *P* < .001; *I* ^2^: 0%). Complications were 7/241 (2.9%) in the RDN + PVI group and 8/237 (3.4%) in the PVI group. The rate of complications between the groups was similar (RR 0.87 [0.33, 2.29], *P* = .77; *I* ^2^: 0%). In the subgroup analysis of paroxysmal AF, RDN + PVI was shown to reduce AF recurrence (RR 0.64 [0.49, 0.82], *P* < .001; *I* ^2^: 0% and HR 0.56 [0.38, 0.82], *P* = .003; *I* ^2^: 0%) compared to PVI alone. RDN + PVI has a moderate certainty of evidence in the reducing AF recurrence with an absolute reduction of 197 fewer per 1000 (from 254 fewer to 124 fewer).

**Conclusion:**

RDN in addition to PVI, is associated with reduced 12‐month AF recurrence and similar procedure‐related complications compared to PVI alone.

## Graphical abstract

There were 568 subjects from 5 studies. RDN+PVI was associated with a lower incidence of AF recurrence compared to PVI alone (RR 0.62 [0.51, 0.76], p<0.001; I2: 0% and HR 0.51 [0.38, 0.70], p<0.001; I2: 0%). The rate of complications between the groups were similar (RR 0.87 [0.33, 2.29], p=0.77; I2: 0%).

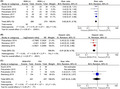

## 1 INTRODUCTION

Atrial fibrillation (AF) and hypertension are highly prevalent diseases that frequently coexist.^1^, ^2^ Hypertension increases the risk of AF,^3^ and uncontrolled hypertension has been shown to be an independent predictor of AF recurrence postablation.^4^ Despite improvement in technologies, the rate of AF recurrence postablation remained high,^5^, ^6^, ^7^ and uncontrolled hypertension poses as an additional problem.

Overactivation of the renin‐angiotensin‐aldosterone system (RAAS) leads to a heightened sympathetic tone, which in turn stimulates renin synthesis.^8^ An enhanced RAAS leads to remodeling along myocardium and vasculature, promoting arterial hypertension and left atrial remodeling.^8^ Renal denervation (RDN) may attenuate the hyperactive sympathetic nervous system which in turn leads to a reduction in RAAS activation and lowering of blood pressure.^9^, ^10^ RDN with or without PVI has been shown to reduce AF burden in hypertensive patients.^11^, ^12^

Hence, RDN as an additional intervention to PVI is potentially advantageous to lower blood pressure (especially in uncontrolled hypertension) and reduce the incidence of AF recurrence. This systematic review and meta‐analysis aimed to assess the latest evidence on the use of RDN + pulmonary vein isolation (PVI) compared to PVI alone for treating atrial fibrillation with hypertension.

## 2 METHODS

### 2.1 Search strategy

**FIGURE 1:**
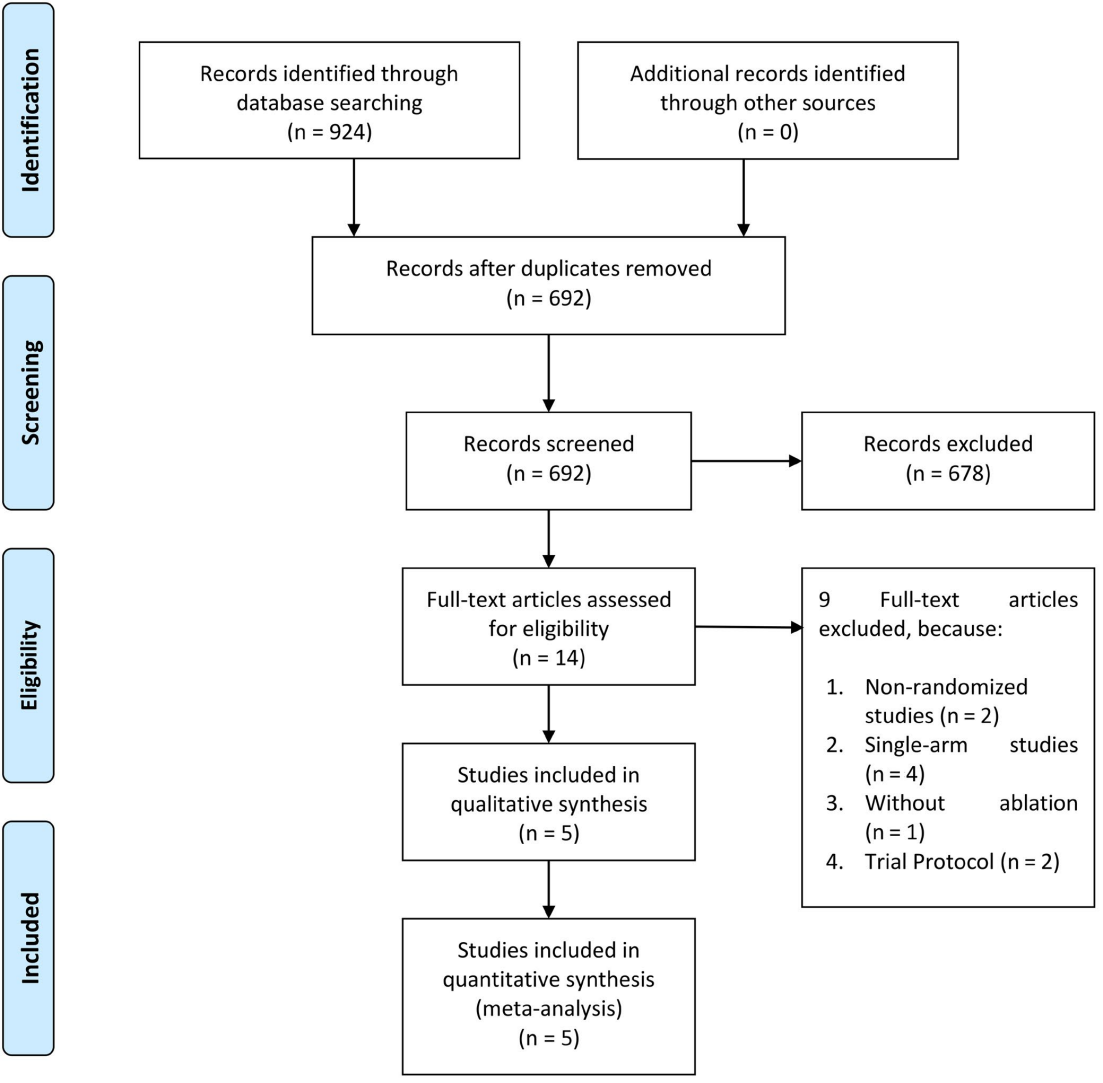
Study flow diagram

We performed a systematic literature search on topics that compare the use of RDN in addition to PVI and PVI alone for hypertensive AF patients undergoing catheter ablation with keywords [“renal denervation” and “ablation” and “atrial fibrillation”] and its synonym from inception up until January 2020 through PubMed, EuropePMC, Cochrane Central Database, ScienceDirect, ProQuest, ClinicalTrials.gov, and hand‐sampling from potential articles cited by other studies. The records were then systematically evaluated using inclusion and exclusion criteria. We also perform hand‐sampling from references of the included studies. Two researchers independently performed the initial search and discrepancies were resolved by discussion. A Preferred Reporting Items for Systematic Reviews and Meta‐Analyses (PRISMA) flowchart of the literature search strategy of studies was presented in Figure 1.

### 2.2 Selection criteria

The inclusion criteria for this study are studies that compare the use of RDN in addition to PVI and PVI alone for hypertensive AF patients undergoing catheter ablation. We include RCTs and exclude observational studies, animal studies, case reports, review articles, and non‐English language articles.

### 2.3 Data extraction

Data extraction and quality assessment were done by two independent authors using a standardized extraction form that includes the first author, the year of publication, study design, sample size, AF recurrence/freedom, complications, paroxysmal AF, age, gender, baseline systolic blood pressure, and follow‐up duration.

The primary outcome was AF recurrence defined as the presence of AF/AFL/AT ≥30 seconds at 12‐month follow‐up and the secondary outcome was procedure‐related complications.

### 2.4 Statistical analysis

To perform the meta‐analysis, we used RevMan version 5.3 software (Cochrane Collaboration). Risk ratios (RRs) were used for pooled effect estimate of dichotomous data, the hazard ratio (HR) was used for pooled effect estimate for HR, and their 95% confidence interval. Inconsistency index (I^2^) test, which ranges from 0% to 100%, was used to assess heterogeneity across studies. A value above 50% or *P* < .10 indicates statistically significant heterogeneity. We used the Mantel‐Haenzsel method to calculate RRs and generic inverse variance to calculate pooled HR using a random‐effects model for meta‐analysis regardless of heterogeneity. Subgroup analysis was performed to evaluate outcome in patients with paroxysmal AF. All *P* values were two‐tailed with a statistical significance set at 0.05 or below. Cochrane risk‐of‐bias tool for randomized trials (Cochrane Collaboration) will be used to assess the risk of bias. The certainty of evidence was assessed using Guideline Development Tool by GRADEpro GDT (McMaster University and Evidence Prime Inc.).

## 3 RESULTS

### 3.1 Study selection and characteristics

We found a total of 924 results, and 692 records remained after the removal of duplicates. 678 records were excluded after screening the title/abstracts. After assessing 14 full‐text for eligibility, we excluded nine because (a) Nonrandomized studies (n = 2), (b) Single‐arm studies (n = 4), (c) Without ablation (n = 1) and (d) Trial Protocol (n = 2). We included five studies in qualitative synthesis and five in meta‐analysis (Figure 1). There were a total of 568 subjects from five studies^12^, ^13^, ^14^, ^15^, ^16^ (Table 1).

**Table 1:** Summary of the included studies

Author	Study design	Sample size (RDN + PVI/PVI)	Inclusion criteria	Primary outcome (recurrence or freedom)	Definition of recurrence	Paroxysmal AF (%)	Age, years (Mean ± SD)	Male (%)	Baseline systolic BP (mm Hg)	Follow‐up period
Steinberg 2019	RCT	302 (154/148)	Paroxysmal AF + Hypertension (≥1 antihypertensive drugs) undergoing cryoballoon PVI	12 months AF/AFL/AT with 3 months blanking period; without AAD after blanking period	AF/AFL/AT ≥30 seconds	100	59 (54‐65) vs 60 (58‐65)	59.1 vs 61.5	150 ± 9 vs 151 ± 9	12 months
Kiuchi 2018	RCT	69 (33/36)	Paroxysmal AF + Uncontrolled hypertension (≥3 antihypertensive drugs) + + CKD + dual‐chamber pacemaker undergoing PVI	12 months AF with 3 months blanking period	AF ≥30 seconds	100	56.8 ± 6.5 vs 58.4 ± 5.1	76 vs 83	142 ± 6 vs 140 ± 6	12 months
Romanov 2017	RCT	76 (39/37)	Paroxysmal/Persistent AF + drug‐resistant hypertension (≥3 antihypertensive drugs) undergoing PVI	12 months AF with 3 months blanking period; without AAD after blanking period	Unclear	38.5 vs 43.2	56 ± 6 vs 56 ± 5	74.3 vs 70.2	163 ± 20 vs 164 ± 16	12 months
Pokushalov 2014	RCT	80 (41/39)	Paroxysmal/Persistent AF + drug‐resistant hypertension (≥3 antihypertensive drugs) undergoing PVI	12 months AF/AFL/AT with 3 months blanking period; without AAD after blanking period	AF/AFL/AT >30 seconds	41.5 vs 46.2	56 ± 6 vs 56 ± 6	75.6 vs 61.5	163 ± 18 vs 164 ± 17	12 months
Pokushalov 2012	RCT	41 (14/13)	Paroxysmal/Persistent AF + drug‐resistant hypertension (≥3 antihypertensive drugs) undergoing PVI	12 months AF/AFL/AT with 3 months blanking period; without AAD after blanking period	AF/AFL/AT >30 seconds	30.8 vs 35.7	57 ± 8 vs 56 ± 9	84.6 vs 71.4	181 ± 7 vs 178 ± 8	12 months

Abbreviations: AAD, antiarrhythmic drugs; AF, atrial fibrillation; AFL, atrial flutter; AT, atrial tachycardia; PVI, pulmonary vein isolation; RCT, randomized controlled trial; RDN, renal denervation.

All studies enrolled AF patients with hypertension. All studies except Steinberg et al enrolled uncontrolled/drug‐resistant hypertension (≥3 antihypertensive drugs). All studies have RDN + PVI as their intervention group, four studies have PVI alone as the control group, and 1 study (Kiuchi et al) has PVI + spironolactone as their control group. Steinberg et al used cryoballoon for PVI, the other studies were unclear. All studies used radiofrequency energy for RDN. All studies assessed AF recurrence for 12 months, the definition for AF recurrence varies but most were AF/AFL/AT ≥30 seconds after initial 3 months blanking period. Steinberg et al and Kiuchi et al only enrolled paroxysmal AF and excluded persistent AF.

### 3.2 Atrial fibrillation recurrence

**FIGURE 2:**
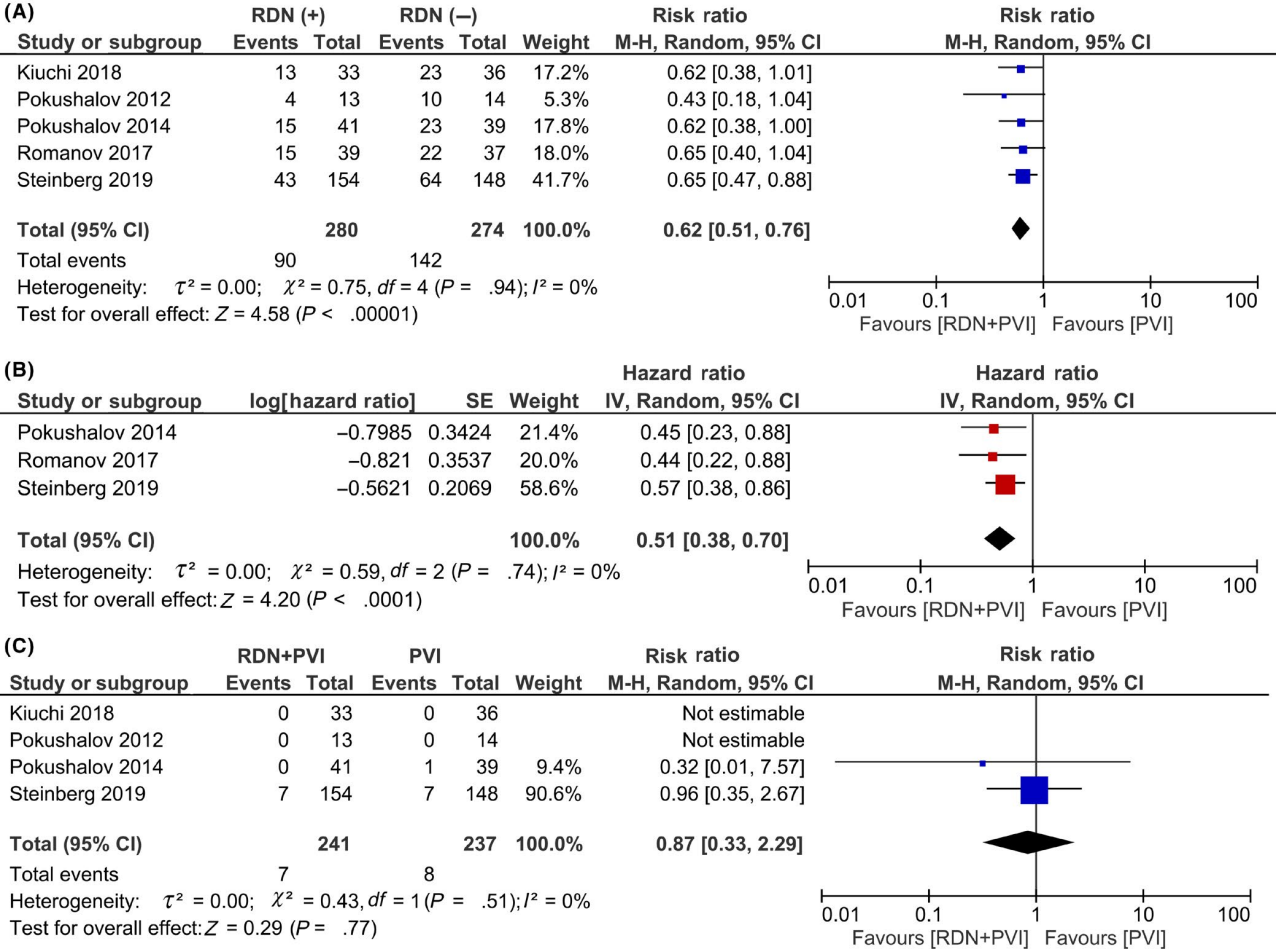
Primary and secondary outcome. RDN + PVI was shown to be associated with reduced AF recurrence (A and B) compared to PVI alone. There was no statistically significant difference between the rate of complication (C) among the two groups. AF, atrial fibrillation; PVI, pulmonary vein isolation; RDN, renal denervation

AF recurrence occurred in 90/280 (32.1%) of the RDN + PVI group and 142/274 (51.8%) of the PVI group. RDN + PVI was associated with a lower incidence of AF recurrence (RR 0.62 [0.51, 0.76], *P* < .001; *I* ^2^: 0%, *P* = .94) (Figure 2A) Three studies also reported the outcome in form of HR, and the pooled HR showed reduced AF recurrence (HR 0.51 [0.38, 0.70], *P* < .001; *I* ^2^: 0%, *P* = .74) in patients receiving RDN + PVI (Figure 2B).

### 3.3 Complications

Complications were 7/241 (2.9%) in the RDN + PVI group and 8/237 (3.4%) in the PVI group out of a total 458 patients from four studies. Procedural complications related to PVI (not clearly defined) were present in seven patients in both groups. There were no complications related to the RDN procedure. The rate of complications between the groups were similar (RR 0.87 [0.33, 2.29], *P* = .77; *I* ^2^: 0%, *P* = .51) (Figure 2C).

### 3.4 Subgroup analysis

**FIGURE 3:**
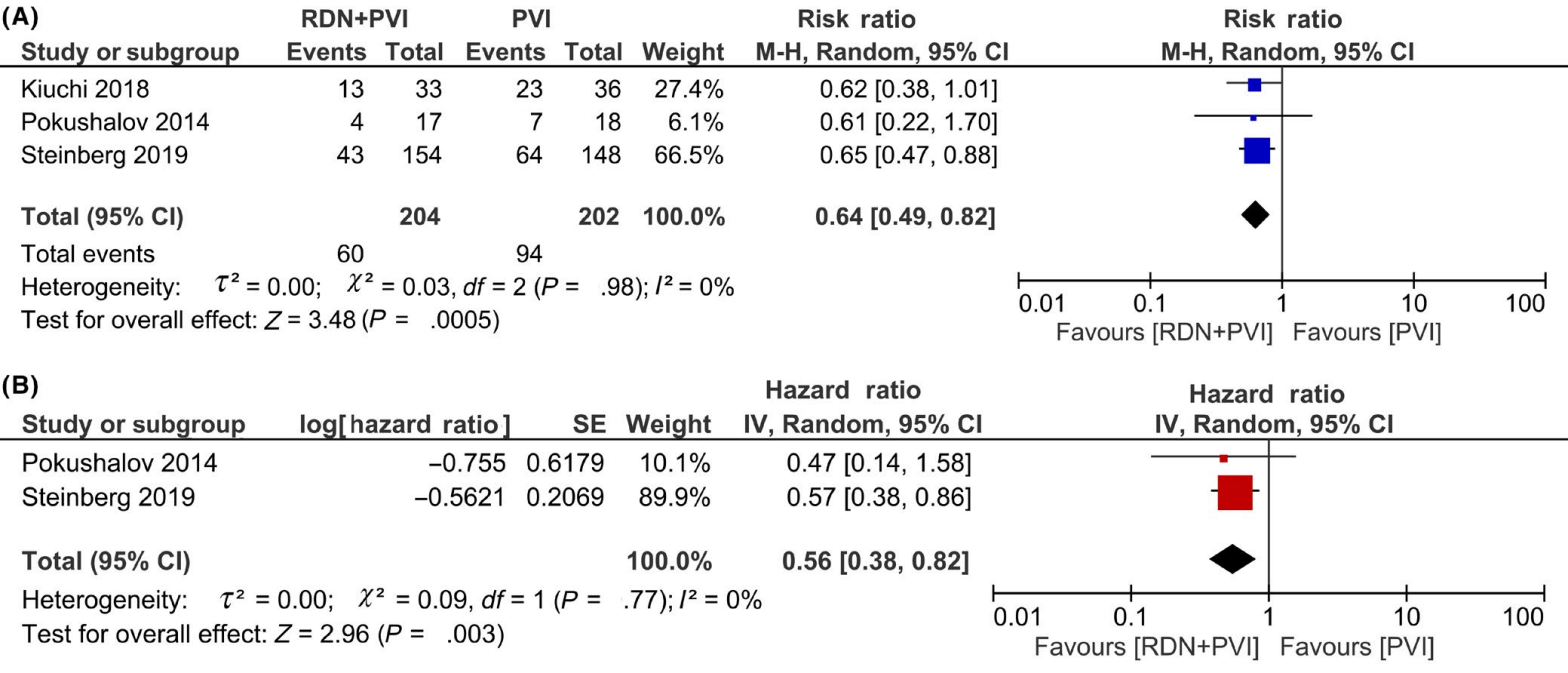
Subgroup analysis on paroxysmal AF. RDN + PVI was shown to be effective in reducing AF recurrence in the paroxysmal AF subgroup (A and B). AF, atrial fibrillation; PVI, pulmonary vein isolation; RDN, renal denervation

In the subgroup analysis of paroxysmal AF, RDN + PVI was shown to reduce AF recurrence (RR 0.64 [0.49, 0.82], *P* < .001; *I* ^2^: 0%, *P* = .98) compared to PVI alone (Figure 3A). Pooled analysis of HR showed that RDN + PVI was associated with reduced AF recurrence (HR 0.56 [0.38, 0.82], *P* = .003; *I* ^2^: 0%, *P* = .77) (Figure 3B).

### 3.5 Risk of bias assessment

**FIGURE 4:**
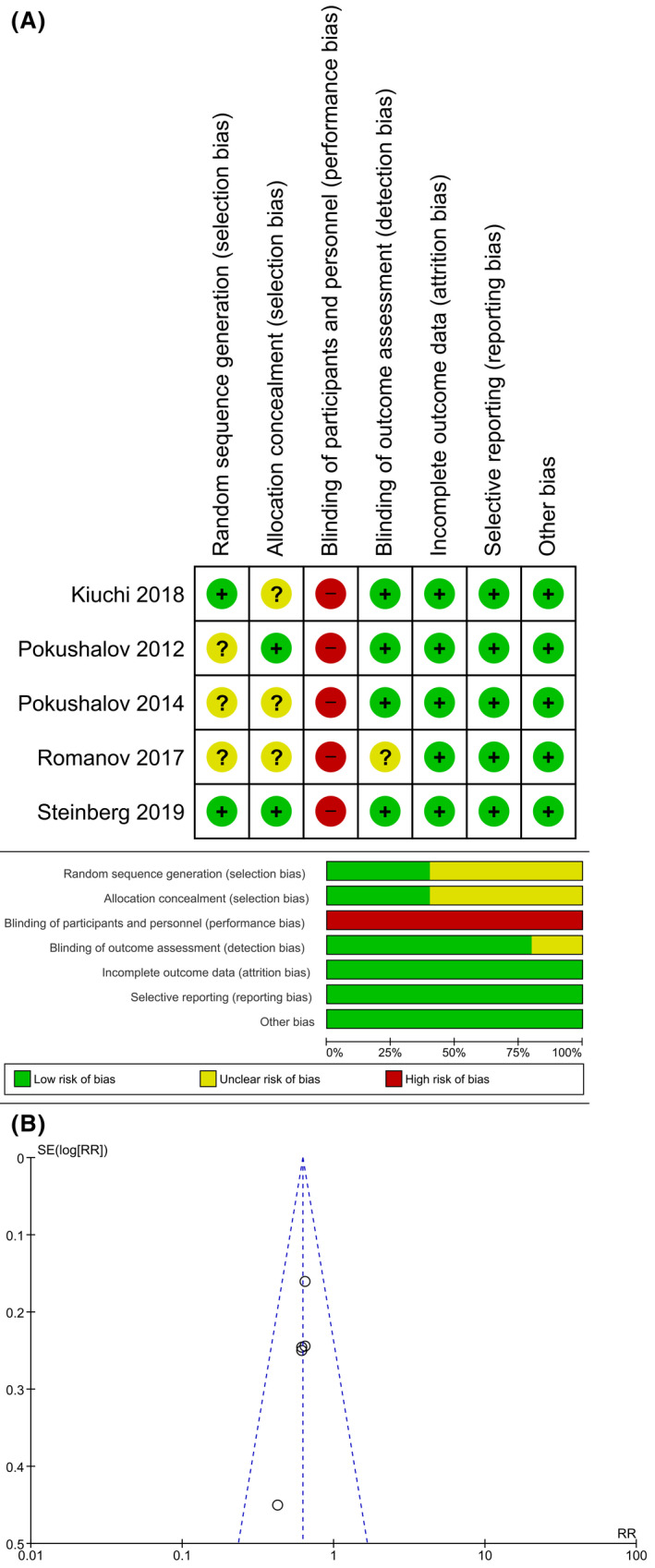
Risk of Bias Assessment. The summary of risk of bias assessment using Cochrane risk‐of‐bias tool for randomized trials was shown in (A). Funnel‐plot analysis demonstrate a relatively symmetrical shape for AF recurrence outcome (B). AF, atrial fibrillation

The risk of bias was mainly due to performance bias because the operators were not blinded to the groups, due to the fact that the same operator performed both PVI and RDN. (Figure 4A) The risk for selection bias was unclear, the method of randomization and allocation concealment was not clearly defined in some studies. The funnel‐plot was symmetrical for the AF recurrence outcome indicating a low‐risk of publication bias. (Figure 4B).

### 3.6 GRADE approach

Grading of Recommendations Assessment, Development, and Evaluation (GRADE) showed a moderate certainty of evidence that RDN in addition to PVI results in the reduction of AF recurrence with an absolute reduction of 197 fewer per 1000 (from 254 fewer to 124 fewer). (Table 2).

**Table 2:** GRADE approach

Certainty assessment							Number of patients		Effect		Certainty	Importance
No of studies	Study design	Risk of bias	Inconsistency	Indirectness	Imprecision	Other considerations	RDN + PVI	PVI only	Relative (95% CI)	Absolute (95% CI)		
AF recurrence												
5	Randomised trials	Serious ^a^	Not serious	Not serious	Not serious	None	90/280 (32.1%)	142/274 (51.8%)	RR 0.62 (0.51‐0.76)	197 fewer per 1000 (from 254 fewer to 124 fewer)	⨁⨁⨁◯ MODERATE	CRITICAL

Abbreviations: AF, atrial fibrillation; CI, confidence interval; PVI, pulmonary vein isolation; RDN, renal denervation; RR, risk ratio.

^a^ Some studies have unclear randomization; Possible performance bias due to impossible blinding of operators

## 4 DISCUSSION

This systematic review and meta‐analysis of RCTs demonstrated that RDN in addition to PVI is associated with reduced 12 months AF recurrence and similar procedure‐related complications compared to PVI alone. The certainty of evidence assessed for the 12 months AF recurrence (moderate certainty) was mainly downgraded due to the risk of bias, nevertheless, inadequate blinding of the healthcare providers may not dramatically impact the effect estimates of RCTs.^17^

The autonomic nervous system has been shown to contribute to the initiation and maintenance of AF^18^, due to associated structural and functional remodeling.^19^, ^20^ Ablating sympathetic nerves present in the renal vasculature reduce systemic and cardiac catecholamine levels, modulates RAAS, and subsequently reduce atrial fibrosis.^21^ In animal models, RDN has been shown to reduce catecholamine levels, reverse atrial structural, and electrical remodeling.^22^, ^23^ In animal ischemic heart failure models, RDN has been shown to reverse structural and electrical remodeling of the atrium along with reduced AF inducibility.^24^ RDN has been though to reduce sympathetic overactivity and arrhythmogenic foci/substrate arising from it.^12^ RDN alone without PVI has been shown to reduce AF burden and increase the quality of life in patients with AF.^11^ Furthermore, RDN in PVI patients was shown to reduce left ventricular septal thickness and posterior wall thickness.^13^, ^16^ Abnormal interventricular septal thickness has been shown to increase the risk of mortality and stroke in AF patients, and the regression of left ventricular hypertrophy has been hypothesized as a therapeutic target.^25^ While shorter diagnosis‐to‐ablation time has been shown to reduce AF recurrence in PVI,^26^ it is not known whether time to RDN will influence its potential to reverse the pathology.

This meta‐analysis showed that RDN + PVI was associated with reduced AF recurrence. Romanov et al reported that RDN in addition to PVI was independently associated with less AF recurrence.^15^ There seemed to be no statistically significant difference between those receiving high‐frequency stimulation compared to those without.^16^

Besides the direct attenuation of sympathetic effect on the atrium, RDN has also been shown to reduce AF recurrence through the reduction of BP in patients with uncontrolled/drug‐resistant hypertension. Romanov et al study showed that the AF burden in RDN + PVI was lower compared to PVI only (2.43% vs 6.95%).^15^ Their study also showed that the blood pressure reduction was associated with less AF burden as demonstrated by the increase in a mean difference of AF burden between the two groups later in follow‐up period. Furthermore, AF recurrence was also shown to be decreased by a HR of 0.9 with each 5 mm Hg decrease in blood pressure.^15^ Kiuchi et al study demonstrated an inverse correlation between blood pressure reduction and AF burden.^12^ This is unsurprising because uncontrolled hypertension (despite ≥3 antihypertensive drugs) has been shown to independently increase the risk of AF recurrence post‐ablation, while controlled hypertension did not affect ablation outcome.^4^ The aforementioned study found that increased atrial size, nonpulmonary vein triggers, and extensive atrial scars were more frequently found in uncontrolled hypertensive group compared to controlled hypertensive, explaining the higher AF recurrence.^4^ Steinberg et al showed that RDN + PVI was associated with reduction in left atrial size which may contribute to a lower AF recurrence.^16^ RDN may help to control blood pressure in patients with uncontrolled hypertension on ≥3 antihypertensive medications,^27^ and as shown by Romanov et al, apparent benefits in terms of AF recurrence were shown with the blood pressure control.

Pokushalov et al (2014) showed that RDN + PVI was effective in persistent AF but not paroxysmal AF, however, this meta‐analysis showed that RDN was also effective in the paroxysmal AF group. Possibly persistent AF has a higher AF recurrence compared to paroxysmal AF in the study, and hence, the sample/event size of paroxysmal AF in Pokushalov et al (2014) study was inadequate to demonstrate significant benefit of RDN. Romanov et al showed that persistent AF was an independent predictor of AF recurrence.^15^

As for the safety aspects of the procedure, there was no single complication related to RDN and the was no statistically significant difference in the number of PVI‐related complications among RDN + PVI and PVI alone. Furthermore, Steinberg et al demonstrated that RDN + PVI was associated with reduced hospitalization due to cardiovascular causes but not mortality compared to PVI alone.^16^ Improvement of renal function in the RDN + PVI group was also demonstrated in Kiuchi et al study.^12^

### 4.1 Clinical implications

RDN in addition to PVI, has a moderate certainty of evidence to reduce AF recurrence in uncontrolled hypertension. There are no additional procedure‐related complications compared to PVI alone. Hence, RDN might be used in addition to PVI in patients with AF and uncontrolled hypertension (≥3 antihypertensive medications). The benefits in patients with hypertension with ≥1 antihypertensive medication(s) has also been demonstrated by a study.

### 4.2 Limitations

The limitation of this systematic review and meta‐analysis is mainly due to publication bias in which positive studies are more likely to be published than negative studies. Although the funnel‐plot analysis did not indicate publication bias, the number of studies is <10 studies. We tried to mitigate the publication bias by only selecting RCTs which are mostly registered on clinicaltrials.gov/other equivalent bodies as opposed to observational studies. These RCTs, if not publish, may report their results in the clinicaltrials.gov, they also showed prespecified outcome that limits the bias. Due to the limited amount of studies, meta‐regression cannot be performed to assess whether age, gender, persistent AF, or blood pressure reduction influence the effect estimate. Most of the studies reported unadjusted HR for the outcome, hence, a pooled estimate of adjusted HR cannot be performed.

## 5 CONCLUSION

RDN in addition to PVI is associated with reduced 12‐month AF recurrence and similar procedure‐related complications compared to PVI alone.

## CONFLICT OF INTEREST

The authors declare no conflict of interests for this article. 

## AUTHORS CONTRIBUTION

R.P conceived and designed the study and drafted the manuscript. R.P and R.V acquired the data and drafted the manuscript. R.P and R.V performed data extraction. R.P, R.V and S.B.R interpreted the data, and performed extensive research on the topic. S.B.R reviewed and performed extensive editing of the manuscript. All authors contributed to the writing of the manuscript. R.P performed the statistical analysis.

## References

[1] Verdecchia P , Angeli F , Reboldi G . Hypertension and atrial fibrillation. Circ Res. 2018;122(2):352–68. 29348255 10.1161/CIRCRESAHA.117.311402

[2] Dzeshka MS , Shantsila A , Shantsila E , Lip GYH . Atrial fibrillation and hypertension. Hypertension. 2017;70(5):854–61. 28893897 10.1161/HYPERTENSIONAHA.117.08934

[3] Gorenek B , Pelliccia A , Benjamin EJ , Boriani G , Crijns HJ , Fogel RI , et al. European Heart Rhythm Association (EHRA)/European Association of Cardiovascular Prevention and Rehabilitation (EACPR) position paper on how to prevent atrial fibrillation endorsed by the Heart Rhythm Society (HRS) and Asia Pacific Heart Rhythm Society (AP). Europace. 2017;19(2):190–225. 28175283 10.1093/europace/euw242 PMC6279109

[4] Santoro F , Di Biase L , Trivedi C , Burkhardt JD , Paoletti Perini A , Sanchez J , et al. Impact of uncontrolled hypertension on atrial fibrillation ablation outcome. JACC Clin Electrophysiol. 2015;1(3):164–73. 29759360 10.1016/j.jacep.2015.04.002

[5] Pallisgaard JL , Gislason GH , Hansen J , Johannessen A , Torp‐Pedersen C , Rasmussen PV , et al. Temporal trends in atrial fibrillation recurrence rates after ablation between 2005 and 2014: a nationwide Danish cohort study. Eur Heart J. 2018;39(6):442–9. 29020388 10.1093/eurheartj/ehx466

[6] Knight BP , Novak PG , Sangrigoli R , Champagne J , Dubuc M , Adler SW , et al. Long‐term outcomes after ablation for paroxysmal atrial fibrillation using the second‐generation cryoballoon. JACC Clin Electrophysiol. 2019;5(3):306–14. 30898232 10.1016/j.jacep.2018.11.006

[7] Pranata R , Vania R , Huang I . Ablation‐index guided versus conventional contact‐force guided ablation in pulmonary vein isolation – systematic review and meta‐analysis. Indian Pacing Electrophysiol J. 2019;19(4):155–60. 31132409 10.1016/j.ipej.2019.05.001 PMC6697487

[8] Dzeshka MS , Shahid F , Shantsila A , Lip GYH . Hypertension and atrial fibrillation: an intimate association of epidemiology, pathophysiology, and outcomes. Am J Hypertens. 2017;30(8):733–55. 28338788 10.1093/ajh/hpx013

[9] Hong M‐N , Li X‐D , Chen D‐R , Ruan C‐C , Xu J‐Z , Chen J , et al. Renal denervation attenuates aldosterone expression and associated cardiovascular pathophysiology in angiotensin II‐induced hypertension. Oncotarget. 2016;7(42). 10.18632/oncotarget.12182 PMC5356522 27661131

[10] Kiuchi MG , Esler MD , Fink GD , Osborn JW , Banek CT , Böhm M , et al. Renal denervation update from the international sympathetic nervous system summit. J Am Coll Cardiol. 2019;73(23):3006–17. 31196459 10.1016/j.jacc.2019.04.015 PMC8559770

[11] Feyz L , Theuns DA , Bhagwandien R , Strachinaru M , Kardys I , Van Mieghem NM , et al. Atrial fibrillation reduction by renal sympathetic denervation: 12 months’ results of the AFFORD study. Clin Res Cardiol. 2019;108(6):634–42. 30413869 10.1007/s00392-018-1391-3 PMC6529371

[12] Kiuchi MG , Chen S , Hoye NA , Pürerfellner H . Pulmonary vein isolation combined with spironolactone or renal sympathetic denervation in patients with chronic kidney disease, uncontrolled hypertension, paroxysmal atrial fibrillation, and a pacemaker. J Interv Card Electrophysiol. 2018;51(1):51–9. 29264729 10.1007/s10840-017-0302-2

[13] Pokushalov E , Romanov A , Corbucci G , Artyomenko S , Baranova V , Turov A , et al. A Randomized comparison of pulmonary vein isolation with versus without concomitant renal artery denervation in patients with refractory symptomatic atrial fibrillation and resistant hypertension. J Am Coll Cardiol. 2012;60(13):1163–70. 22958958 10.1016/j.jacc.2012.05.036

[14] Pokushalov E , Romanov A , Katritsis DG , Artyomenko S , Bayramova S , Losik D , et al. Renal denervation for improving outcomes of catheter ablation in patients with atrial fibrillation and hypertension: early experience. Hear Rhythm. 2014;11(7):1131–8. 10.1016/j.hrthm.2014.03.055 24691452

[15] Romanov A , Pokushalov E , Ponomarev D , Strelnikov A , Shabanov V , Losik D , et al. Pulmonary vein isolation with concomitant renal artery denervation is associated with reduction in both arterial blood pressure and atrial fibrillation burden: data from implantable cardiac monitor. Cardiovasc Ther. 2017;35(4):e12264. 10.1111/1755-5922.12264 28423234

[16] Steinberg JS , Shabanov V , Ponomarev D , Losik D , Ivanickiy E , Kropotkin E , et al. Effect of renal denervation and catheter ablation vs catheter ablation alone on atrial fibrillation recurrence among patients with paroxysmal atrial fibrillation and hypertension. JAMA. 2020;323(3):248. 31961420 10.1001/jama.2019.21187 PMC6990678

[17] Moustgaard H , Clayton GL , Jones HE , Boutron I , Jørgensen L , Laursen DRT , et al. Impact of blinding on estimated treatment effects in randomised clinical trials: meta‐epidemiological study. BMJ. 2020 l6802 10.1136/bmj.l6802 31964641 PMC7190062

[18] Chen P‐S , Chen LS , Fishbein MC , Lin S‐F , Nattel S . Role of the autonomic nervous system in atrial fibrillation. Circ Res. 2014;114(9):1500–15. 24763467 10.1161/CIRCRESAHA.114.303772 PMC4043633

[19] Krogh‐Madsen T , Abbott GW , Christini DJ . Effects of electrical and structural remodeling on atrial fibrillation maintenance: a simulation study. McCulloch AD, ed. PLoS Comput Biol. 2012;8(2):e1002390. 22383869 10.1371/journal.pcbi.1002390 PMC3285569

[20] Pranata R , Yonas E , Vania R . Prolonged P‐wave duration in sinus rhythm pre‐ablation is associated with atrial fibrillation recurrence after pulmonary vein isolation—a systematic review and meta‐analysis. Ann Noninvasive Electrocardiol. 2019;24(5). 10.1111/anec.12653 PMC6931719 30983090

[21] Waldron NH , Fudim M , Mathew JP , Piccini JP . Neuromodulation for the treatment of heart rhythm disorders. JACC Basic to Transl Sci. 2019;4(4):546–62. 10.1016/j.jacbts.2019.02.009 PMC6712352 31468010

[22] Wang X , Huang C , Zhao Q , Huang H , Tang Y , Dai Z , et al. Effect of renal sympathetic denervation on the progression of paroxysmal atrial fibrillation in canines with long‐term intermittent atrial pacing. Europace. 2015;17(4):647–54. 10.1093/europace/euu212 25349225

[23] Linz D , van Hunnik A , Hohl M , Mahfoud F , Wolf M , Neuberger H‐R , et al. Catheter‐based renal denervation reduces atrial nerve sprouting and complexity of atrial fibrillation in goats. Circ Arrhythmia Electrophysiol. 2015;8(2):466–74. 10.1161/CIRCEP.114.002453 25713217

[24] Yamada S , Fong M‐C , Hsiao Y‐W , Chang S‐L , Tsai Y‐N , Lo L‐W , et al. Impact of renal denervation on atrial arrhythmogenic substrate in ischemic model of heart failure. J Am Heart Assoc. 2018;7(2). 10.1161/JAHA.117.007312 PMC5850156 29358197

[25] Apostolakis S , Sullivan RM , Olshansky B , Lip GYH . Left ventricular geometry and outcomes in patients with atrial fibrillation: The AFFIRM Trial. Int J Cardiol. 2014;170(3):303–8. 24315343 10.1016/j.ijcard.2013.11.002

[26] Pranata R , Chintya V , Raharjo SB , Yamin M , Yuniadi Y . Longer diagnosis‐to‐ablation time is associated with recurrence of atrial fibrillation after catheter ablation—Systematic review and meta‐analysis. Journal of Arrhythmia. 2020;36(2):289–94. 32256876 10.1002/joa3.12294 PMC7132183

[27] Kandzari DE , Böhm M , Mahfoud F , Townsend RR , Weber MA , Pocock S , et al. Effect of renal denervation on blood pressure in the presence of antihypertensive drugs: 6‐month efficacy and safety results from the SPYRAL HTN‐ON MED proof‐of‐concept randomised trial. Lancet. 2018;391(10137):2346–55. 29803589 10.1016/S0140-6736(18)30951-6

